# Therapeutic efficacy of platinum/etoposide regimens in the treatment of advanced poorly differentiated neuroendocrine carcinomas of the lung: A retrospective analysis

**DOI:** 10.3389/fendo.2023.1065599

**Published:** 2023-01-30

**Authors:** Ivana Puliafito, Federico Chillari, Alessandro Russo, Ornella Cantale, Dorotea Sciacca, Luigi Castorina, Cristina Colarossi, Tindara Franchina, Maria Paola Vitale, Giuseppina Rosaria Rita Ricciardi, Vincenzo Adamo, Francesca Esposito, Dario Giuffrida

**Affiliations:** ^1^ Medical Oncology Unit, Istituto Oncologico del Mediterraneo SpA, Viagrande, Italy; ^2^ Department of Medical Oncology, Papardo Hospital & University of Messina, Messina, Italy; ^3^ Nuclear Medicine, Istituto Oncologico del Mediterraneo SpA, Viagrande, Italy; ^4^ Pathology Unit, Istituto Oncologico del Mediterraneo SpA, Viagrande, Italy; ^5^ Hospital Pharmaceutical Unit, ASL Roma 6, Ariccia, Italy

**Keywords:** platinum regimens, platinum/etoposide schedules, neuroendocrine neoplasms (NENs), neuroendocrine carcinomas (NECs), neuroendocrine tumors (NETs), poorly differentiated lung NECs

## Abstract

**Background:**

Lung neuroendocrine neoplasms (NENs) are rare malignancies developed from bronchial mucosa. Because of its rarity and complex histopathology, there is limited data on the role of chemotherapy in this subset of tumors. Few studies regarding the treatment of poorly differentiated lung NENs, known as neuroendocrine carcinomas (NECs), are available and many limits are detectable as heterogeneity of tumor samples including different origins and different clinical behaviors, moreover, no evidence of therapeutic advances have been achieved along the last thirty years.

**Method:**

We performed a retrospective analysis of 70 patients affected by poorly differentiated lung NECs: half of patients underwent a first line therapy with a combination of cisplatin plus etoposide; the remaining patients receiving carboplatin instead of cisplatin, plus etoposide. Results: In our analysis, the outcomes of patients treated with either cisplatin or carboplatin schedule are similar in terms of ORR (44% versus 33%), DCR (75% versus 70%), PFS (6.0 versus 5.0 months) and OS (13.0 versus 10 months). Median number of chemotherapy cycles was 4 (range 1-8). The 18% of patients required a dose reduction. Main toxicities reported were hematological (70.5%), gastrointestinal (26.5%) and fatigue (18%).

**Conclusion:**

Survival rate in our study suggests that high grade lung NENs are characterized by an aggressive behavior and a poor prognosis, despite the treatment with platinum/etoposide according to available data. Clinical results of present study contribute to strengthen available data on the usefulness of platinum/etoposide regimen in the treatment of poorly differentiated lung NENs

## 1 Introduction

The term neuroendocrine neoplasms (NENs) identifies a group of heterogeneous tumors characterized by the presence of neurosecretory granules. These tumors also show a characteristic histology and immunoprofile. NENs potentially affect multiple organs, showing pathological features proper of the neuroendocrine category (1). In November 2017, the World Health Organization (WHO) International Agency for Research of Cancer (IARC) introduced a new classification for NENs, allowing for the unification of pre-existing classification concepts, despite organ-specific differences in classification criteria, tumor biology, and prognostic factor (2). The new classification encompasses the term NENs for both differentiated neuroendocrine tumors (NETs) and poorly differentiated neuroendocrine carcinomas (NECs), in which NEC is indicative of high-grade malignant histology and biologic behavior. NENs derived from the pulmonary and digestive systems can be used as a model of difference in biological aggressiveness and response in medical therapy observed in NETs and NECs (2). Site of origin and embryological origin identify different NENs. Lung NENs develop from bronchial mucosa, meaning they can be labelled as foregut derivatives. About 25% of primary lung cancers are NENs, including small cell lung cancer (SCLC), which represents 20% of all lung cancers. The remaining 5% are composed of large cell neuroendocrine cancers (LCNEC, 3%), typical carcinoids (TCs, 1,8%), and atypical carcinoids (ACs, 0,2%). TCs and ACs are well-differentiated and correspond to NET, while LCNEC and SCLC are poorly differentiated and correspond to NEC (2). SCLC is the most malignant pole in the spectrum of lung NECs.

In the present study, prognostic relevance was established using three parameters: mitotic rate, Ki-67 labeling index, and presence or absence of necrosis. Mitotic rate is defined as mitoses per mm^2^ area; in lung NENs, it is expressed as mitoses within an area of 2mm^2^. The Ki-67 protein expression is a marker of cellular proliferation, measured by immunolabelling, which allowed us to analyze the number of cells expressing it. Tumor grade was defined as the proliferation degree expressed through the mitotic rate, alongside necrotic features. Indeed, the mitotic rate expresses mitosis to tumor area ratio (mitoses per 2mm^2^, and not per 10 Hpf, see **Table 1**). Furthermore, these four histological identities are framed by the following morphological characteristics: chromatin appearance, nucleoli presence/absence, tumor cells dimension, and shape. TC showed polygonal cells, no necrosis, and <2 mitoses/2mm^2^. AC showed a raise in mitotic rate to 2-10 mitoses/2mm^2^ and/or necrosis foci (clusters). LCNEC showed large cells, fence architecture, prominent nucleoli, condensed chromatin, even higher mitotic rate (>10 mitoses/2mm^2^), and extended necrosis. Finally, SCLC showed oval spindles and small cells, the mitotic rate increased to >10 mitoses/2mm^2^ and there was finely dispersed chromatin, evident nucleoli, and map-like necrosis (**Table 1**) (3).

**Table 1:** 2015 WHO classification of lung NENs^3^.

Differentiation	Grade	Mitotic Rate
Well differentiated	Low grade	< 2 mitoses per 2 mm^2^ and no necrosis
	Intermediate grade	< 2 mitoses per 2 mm^2^ and no necrosis
Poorly differentiated	High grade	> 11 mitoses per 2 mm^2^

TC, typical carcinoid; AC, atypical carcinoid; SCLC, small cell lung cancer; LCNEC, large cell neuroendocrine carcinoma.

Ki-67 is used in the classifications of gastroenteropancreatic (GEP)-NENs. It has not been fully embodied in lung NENs classification yet, due to conflicting data concerning the accuracy of the technique (4, 5). The Ki-67 index in lung NENs was useful to identify high grade LCNEC and SCLC from TC/ACs (6). A Ki-67 greater than 50% is typical of high-grade NECs, while a Ki-67 lesser than 20% is for TCs and ACs (<5% for TCs compared to 5-20% for ACs) (6, 7).

The embodiment of WHO classification in actual clinical practice might not be as smooth as expected for many reasons, including the pathology diagnostic rate, inter-observer variability, and assessment of morphological parameters inevitably bound to histological samples, making it difficult to carry out a diagnosis on small biopsies or cytological samples (8).

The treatment of poorly differentiated lung NETs is based on platinum regimens, but there are limited data that demonstrate its efficacy (9, 10). Many available studies have limitations, such as the heterogeneity of tumor samples including different origins and different clinical behaviors. No evidence of therapeutic advances has been achieved over the last thirty years.

Our goal is to evaluate the therapeutic efficacy of platinum/etoposide schedules in the treatment of poorly differentiated lung NECs treated in two Sicilian Institutions during the last decade through a retrospective analysis of 70 patients.

## 2 Material and methods

### 2.1 Population

This retrospective analysis was compliant with all relevant ethical regulations involving human participants and was approved by the Istituto Oncologico del Mediterraneo Institutional Review Board (project ID code: n_1 of 24.09.2015) and by the Papardo Institutional Review board. Signed informed consent was obtained for each patient.

We performed a retrospective revision of the medical record in the database of the IOM Oncologic Unit and Papardo Hospital Oncologic Unit, regarding 70 patients with histological confirmation of LCNEC or SCLC (according to the WHO 2015 classification), advanced (stage IIIB) or metastatic (stage IV) (11, 12), treated with a combination of cisplatin/carboplatin and etoposide as first-line therapy. Patients had measurable lesions according to RECIST criteria (version 1.1) (13). For all patients, the stage of the lesion was established according to TNM classification by the American Joint Committee on Cancer (AJCC), version VIII (11), and Extensive Disease of Veteran administration lung study group (VALSG) (12).

Medical records collected clinical data of patients, such as patient main characteristics (eg. age, sex, and clinical history), as well as information regarding disease and related treatment. Clinical data were collected over a decade, between September 2006 and December 2016.

### 2.2 Treatment plan, response assessment, and statistical analysis

Half of the patients underwent first line therapy with a combination of cisplatin plus etoposide: etoposide was administered 130 mg/m^2^/day in days 1-2-3 and cisplatin 45 mg/m^2^ on days 2→3, administered every 21 days by intravenous infusion. The remaining patients received carboplatin instead of cisplatin. The choice to administer cisplatin vs carboplatin was based on age, patient characteristics, disease, and comorbidities. Carboplatin was preferred in: *i.* patients aged > 75 years; *ii.* patients with poor ECOG performance status (PS); *iii.* patients with subnormal kidney function (because of major nephrotoxicity of cisplatin, kidney function was evaluated before starting the chemotherapy); and *iv.* patients with adequate bone marrow reserve, due to major risks of hematological toxicity related to the use of carboplatin. Diffuse bone metastases could be associated with minor bone marrow reserve; in these cases, cisplatin has been preferred to carboplatin. The study did not have the expected randomization, as based on the above, 50% of patients received cisplatin while the other 50% received carboplatin. It was a coincidence that the population was been divided in half.

A multidisciplinary team evaluated the clinical status of each patient. According to RECIST criteria 1.1, tumor response was assessed as Complete Response (CR), Partial Response (PR), and Overall Response Rate (ORR) was a sum of CR and PR. Disease Control Rate (DCR) is defined as the Stable Disease (SD) plus ORR. Survival analysis was conducted evaluating progression free survival (PFS), defined as the time from the beginning of first line platinum/etoposide chemotherapy to progression of disease and/or death for any cause, and overall survival (OS), defined as the time from the beginning of first line platinum/etoposide chemotherapy till death. PFS and OS were calculated through the Kaplan-Meier method. Survival curves were compared using log-rank test. The hazard Ratio (HR) was estimated by using Cox regression analysis. Proportions were compared with the non-parametric *χ* ^2^ test.

## 3 Results

Between September 2006 and December 2016, a total of 70 patients with lung NECs treated with platinum/etoposide chemotherapy were included in the analysis. Patients did not receive a previous chemotherapy regimen of systemic therapy for advanced/metastatic disease. Patient characteristics included in the analysis are summarized in **Table 2**. The median age was 67 years (range 45-80). Patients were 81% male and 19% female, with ECOG PS of 0-2 in 97% of patients and 3 in 3%. Patients present a tumor stage distribution at baseline as follows: stage IV in 91% of patients and stage III in 9% of patients. Baseline symptoms were present in 63/70 of patients: the most common symptoms were cough (25%), pain (35%), and dyspnea (36.5%). Cisplatin/etoposide scheme was used in 50% of patients, while the carboplatin/etoposide scheme was used in the remaining 50% of the population.

**Table 2:** Baseline clinicopathological patients’ features.

Variables	No. (tot=70)	
**Age (median)**	67 (range 45-80)	
**Gender**	Male	57 (81%)
	Female	13 (19%)
**Histology**	SCLC	58 (82%)
	LCNEC	12 (18%)
**Tobacco use**	Yes	62 (88.8%)
	No	3 (3.7%)
	Former smoker	5 (7.4%)
**Stage**	III	6 (9%)
	IV	64 (91%)
**Metastatic site at baseline**	Liver	18 (26%)
	Brain	8 (12%)
	Bones	22 (32%)
	>3 sites	30 (43%)
**Previous surgery**	Yes	7 (10%)
	No	63 (90%)
**ECOG PS at baseline**	0	18 (25%)
	1	36 (51%)
	2	15 (21%)
	3	1 (3%)

**Figure 1:**
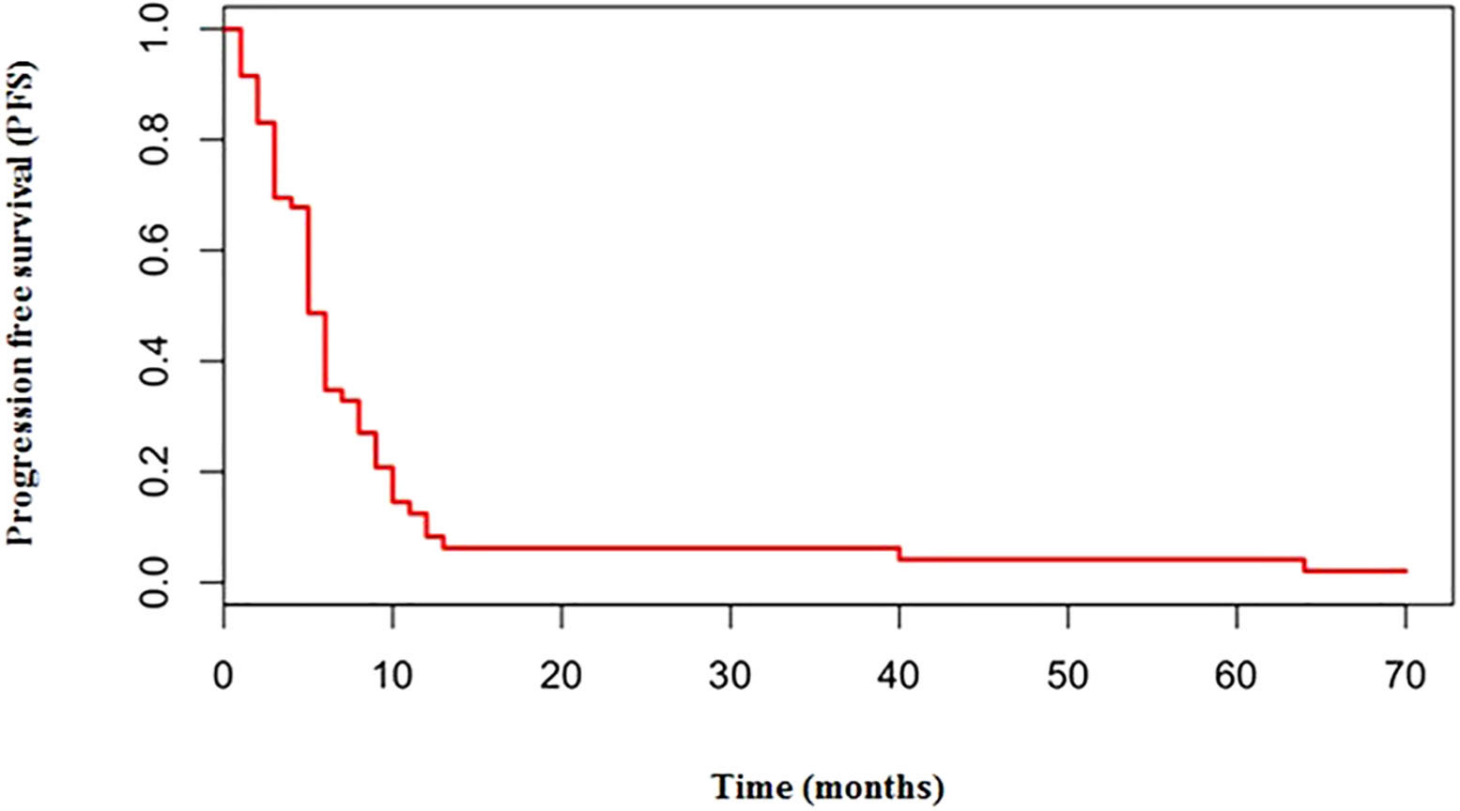
Median PFS in overall population treated with platinum/etoposide mPFS: 5 months.

**Figure 2:**
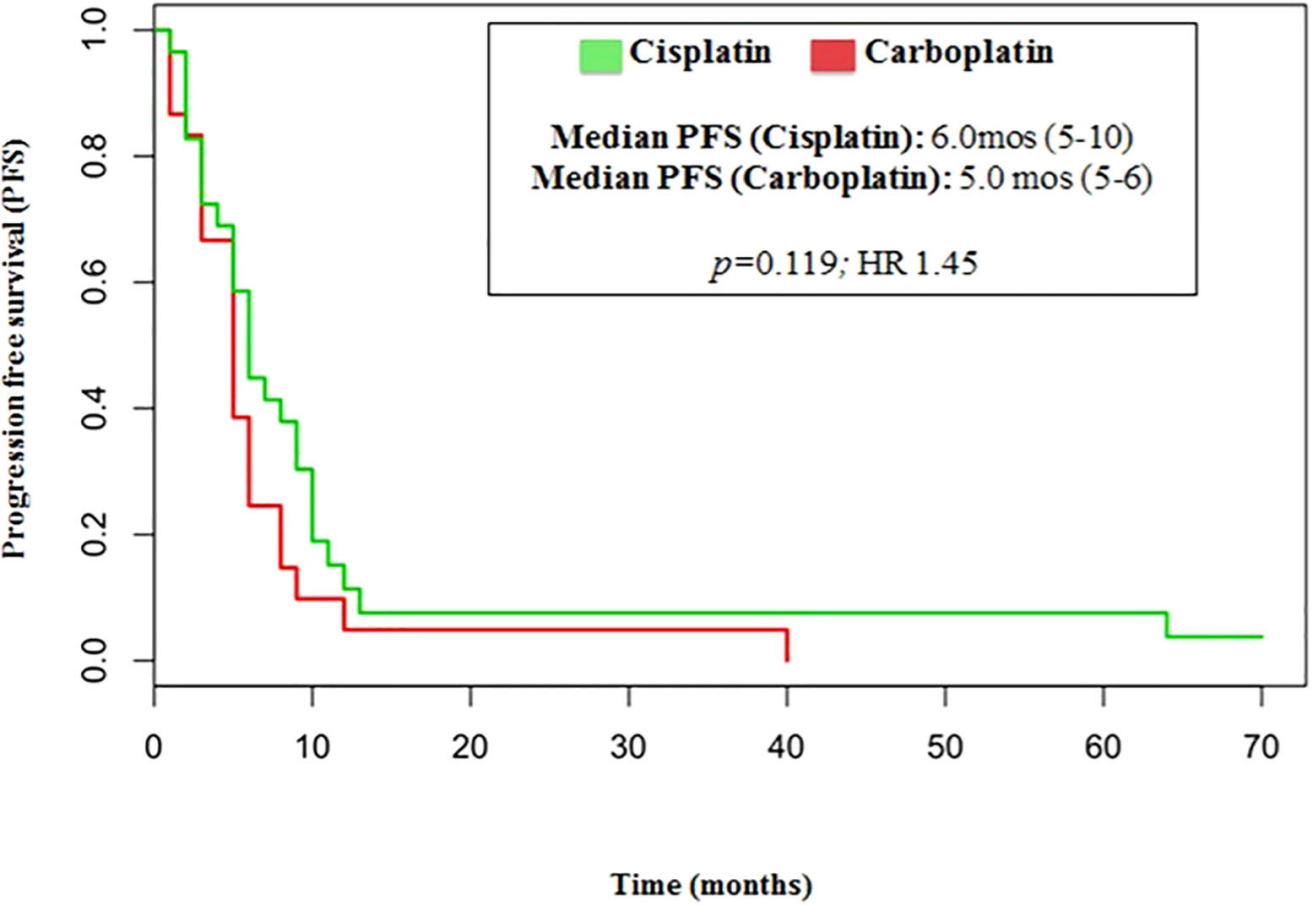
Median PFS related to cisplatin/etoposide scheme and carboplatin/etoposide scheme.

**Figure 3:**
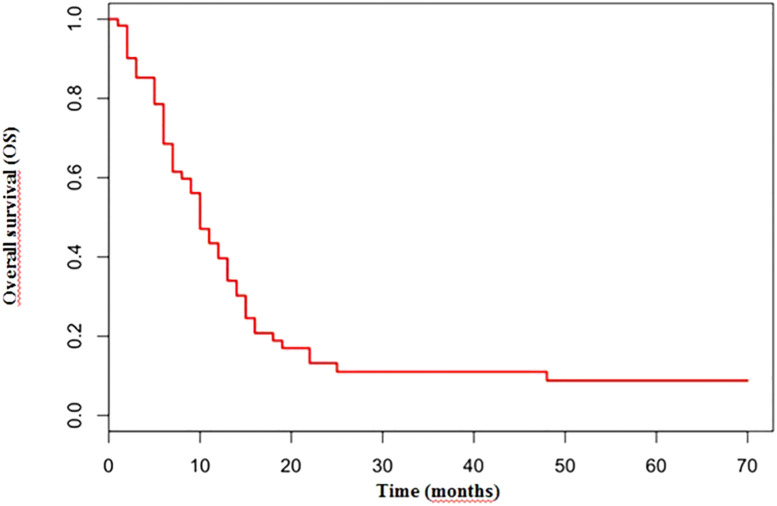
Median OS in overall population treated with platinum/etoposide mOS: 10 months.

The median number of chemotherapy cycles was 4 (range 1-8). In total, 18% of patients required a dose reduction. The main toxicities reported were hematological (70.5%), gastrointestinal (26.5%) and fatigue (18%). ORR was 39% (5% CR) and DCR was 73%. The proportion of patients receiving second line treatment after progression was 44%. In total, 15% of patients were still alive when the present analysis was edited. PFS was 5.0 months (**Figure 1**). Median PFS was respectively 6 months in the cisplatin/etoposide group and 5.0 months in the carboplatin/etoposide group. No significant differences were reported between the two groups (p=0, 119; HR 1.45) (**Figure 2**). Median OS was 10.0 months, (**Figure 3**) scraping 39% of the population (CI 95%, 28-54) and 11% (CI 95%, 5-23) at 12 and 24 months, respectively.

**Figure 4:**
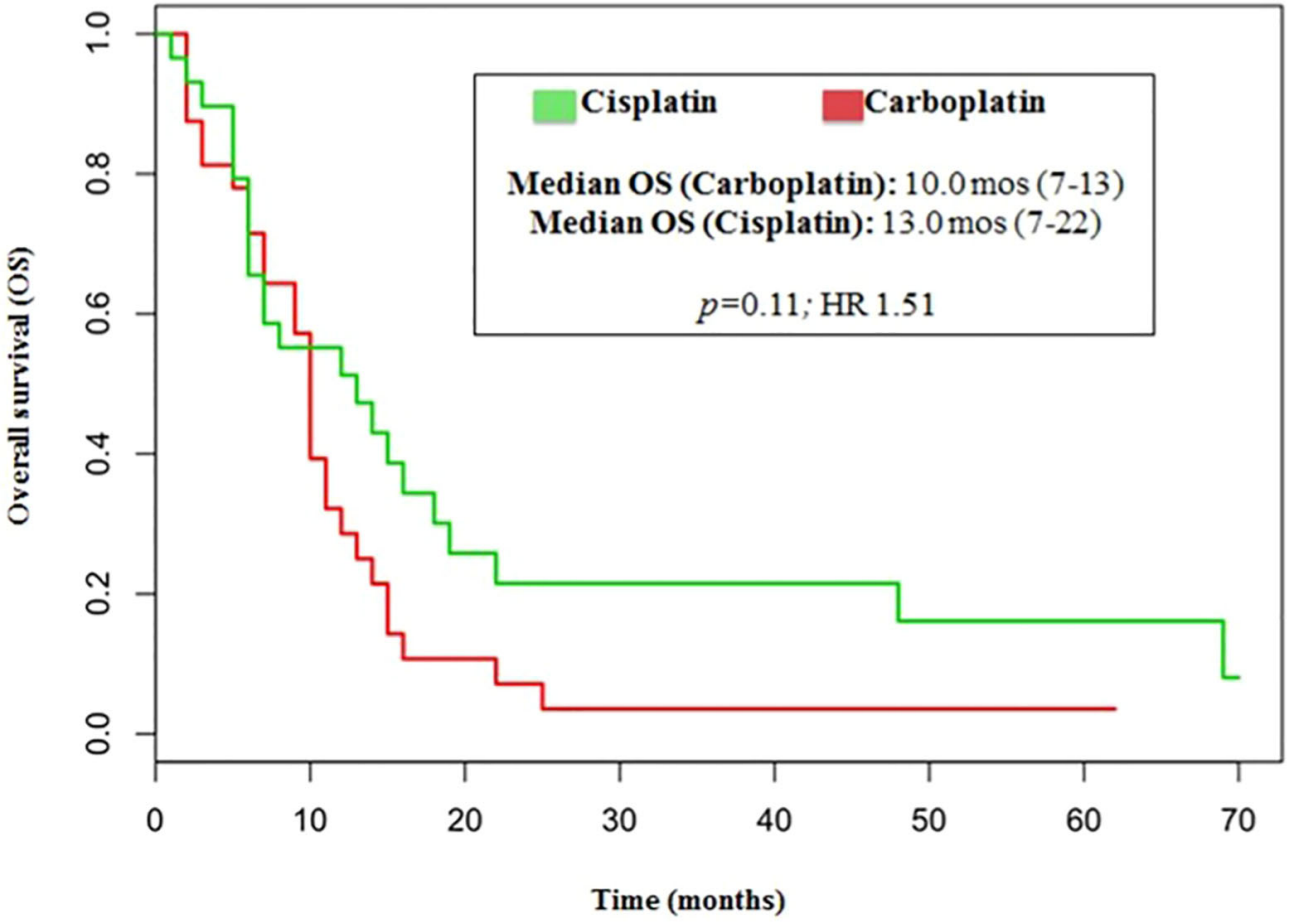
Median OS related to cisplatin/etoposide scheme and carboplatin/etoposide scheme.

No significant differences were reported in terms of OS between cisplatin and carboplatin groups (P=0.11; HR 1.51) (**Figure 4**).

No significant difference was reported in terms of median PFS (p=0.082; HR 0.95) and OS (p=0.81; HR 1.09) between SCLC and LCNEC histological subgroups, according to previous reports.

## 4 Discussion

In 1991 Moertel et al. (9), published the results of a study that represents, even today, the main reference in this subset of patients. In their study, a treatment scheme with etoposide 130mg/m^2^ G1-3 plus cisplatin 45 mg/m^2^ G 2-3 every 4 weeks was evaluated in 45 patients with metastatic NENs. Among the 45 eligible and evaluable patients, 13 had well-differentiated carcinoid tumors, 14 had well-differentiated islet cell carcinomas, and 18 had anaplastic NENs.The overall regression was 67% and the complete regression rate of 17% in patients with anaplastic NENs was 19 months.

In a retrospective study, Mitry et al. (10) assessed the efficacy of chemotherapy based on etoposide and cisplatin regimens, structured as a combination of etoposide 100 mg/m^2^/day in days 1-2 and cisplatin 100 mg/m^2^ on day 1, given in two hours through intravenous infusion, administered every 21 days. The study evaluated a total of 53 patients. Among these, only 7 had a lung NEN. The overall response rate ORR was 41.5% among patients with a poorly differentiated tumor and 9.1% among patients with a well-differentiated tumor, with a not significant difference (P=0.09). Median OS was 17.6 months in well differentiated tumor and 15 months in poorly differentiated tumors.

Small retrospective studies suggest that the outcome in LCNEC patients is similar to SCLC patients after being treated with the appropriate regimen. However, different national guidelines, including NCCN, recommend treating LCNEC patients with NSCLC-like regimens (14–16).

A small phase II prospective study evaluated 42 patients with LCNEC, reported after treatment with cisplatin 80mg/m^2^, G1 and etoposide 100 mg/m^2^ G1- 3 q21 a median PFS of 5.2 monthsand a median OS of 7.7 months (17). Our analysis confirms the poor prognosis of lung NETs treated with first line platinum/etoposide regimen. PFS (5.0 months) and OS (10.0 months) results are similar to those of clinical studies in the wider literature. No significant difference was reported in terms of PFS (6.0 vs 5.0 months, p=0.882, HR 0.95) or OS (10.0 months in both groups, p=0.81, HR 1.09) between LCNEC and SCLC according to previous reports.

The survival rate in our study suggests that high grade lung NECs are characterized by an aggressive behavior and a poor prognosis, despite the treatment with platinum/etoposide according to results shown in studies by Moertel et al. (9) and Mitry et al. (10).

In our analysis, the outcomes of patients treated with either cisplatin or carboplatin schedule are similar in terms of ORR (44% vs 33%, respectively; p=0.146), DCR (75% vs 70%, respectively; p=0.526), PFS (6.0 vs 5.0months, respectively; p=0.110, HR 1.45) and OS (13.0 vs 10 months, respectively; p=0.11, HR 1.51).

The present study has some potential limitations. First, it involved a low number of patients (n=70). Moreover, in 18% of treated patients, a reduced dose of chemotherapy was necessary due to poor tolerability. This introduced bias that could have influenced final results. Finally, this was a retrospective analysis, which meant we were unable to randomize patients. In future research a prospective study will be required to provide more information.

New therapeutic strategies have been evaluated for patients with lung NECs, for instance the use of immunotherapy in SCLC pretreated patients, already supported by promising results provided by Checkmate 032 (18) and KEYNOTE 028 (19), after treatment or maintenance (20). Moreover, PARP inhibitors have been recently reported to show interesting activity in Extensive Disease (ED)-SCLC.

## 5 Conclusion

This retrospective study investigated the effectiveness of a platinum/etoposide regimen in a subset of 70 subjects affected by poorly differentiated lung NECs. The outcomes of patients treated with either cisplatin or carboplatin schedule were similar in terms of ORR (44% versus 33%), DCR (75% ersus 70%), PFS (6.0 versus 5.0 months), and OS (13.0 versus 10 months).

These results contribute to strengthen available data on the usefulness of platinum/etoposide regimen in this subset of patients.

## Data availability statement

The original contributions presented in the study are included in the article/Supplementary Material. Further inquiries can be directed to the corresponding authors.

## Ethics statement

This retrospective analysis was compliant with all relevant ethical regulations involving human participants and was reviewed and approved by the Istituto Oncologico del Mediterraneo Institutional Review Board (project ID code: n_1 of 24.09.2015) and by Papardo Institutional Review board. Written informed consent was obtained for each patient.

## Author contributions

IP wrote the manuscript. FC, OC, LC, VA, DG provided substantial contributions to study conception. AR, DS, CC, TF, MPV and GRRR helped to conduct the study, providing patient assistance, and undertaking drug preparation and data analysis. FE revised the manuscript. All authors have read and agreed to the published version of the manuscript.
